# Supplementary material for: Histone lactylation: A new epigenetic mark in the malaria parasite Plasmodium
Source: PLoS Genet. 2025 Dec 19;21(12):e1011991. doi: 10.1371/journal.pgen.1011991 (PMC12742760; doi:10.1371/journal.pgen.1011991)
Supplement: Attachment — Submitted filename: Response to reviewers LETTER.pdf [file pgen.1011991.s022.pdf]

Point-by-point responses to all reviewer comments are provided in blue text below.

<u>Reviewer #1:</u>

The topic of the paper is highly interesting, and the finding of lactylation of histones and other proteins in Plasmodium species is an important and new finding. However, the study is mostly descriptive, and the paper severely suffers from far too low numbers of replicate analyses, rendering statistically relevant comparisons fairly unreliable.

We appreciate the positive assessment of the manuscript's novelty and interest.

In general, we would dispute the 'low numbers' of replicate analyses: almost all experiments except the purely qualitative ones like immunofluorescence images – and *all* of those for which statistical significance is claimed – were conducted at least in triplicate, as is standard in the field.

Major comments:

1. In most or all experiments with Western blot analyses, an insufficient number of biological replicates is shown. For instance, only two datapoints per condition is available in several figures (e.g. figure 2, figure 3, but also other figures). This is obviously not acceptable and the number of replicates needs to be upscaled.

For instance, in figure 3E, absolutely nothing can be concluded when comparing the control vs the lactate condition. In general, for this type of analysis an absolute minimum of 5 to 6 datapoints per condition (and per time point) is required, in particular since it is clear that there is variability present (although with the number of data points provided, it is even hard to get an idea of the variability). This is the absolute minimum to obtain somewhat reliable statistical comparisions.

Throughout Figure 1 and Figure 3A-D, we have actually quantified 3-4 replicates for all the *P. falciparum* and *P. knowlesi* blots (plus 7 separate mice for the blot of *P. yoelii* in Figure 1). These are the only places where <u>statistically significant changes</u> in histone lactylation, at a single timepoint, are reported. Since western blotting is, by its nature, only semi-quantitative (and is frequently shown in published work as a purely qualitative readout, without densitometric quantification), we consider this to be sufficient. It is actually rare to see western-blot data quantified from 6 biological replicate experiments! A selection of published examples to the contrary includes:

- The original Nature paper discovering histone lactylation in 2019 (doi.org/10.1038/s41586-019-1678-1). Many western blots are shown to demonstrate changes in histone lactylation, either unquantified and simply stated to be 'representative of 3 independent blots' (see Fig 2), or quantified from maximally 2 or 3 independent blots (see Fig 3).
- An example in *Plasmodium*: Nat Commun. 2025 Mar 6;16(1):2259 'Elevated NAD^+ drives Sir2A-mediated GC-beta deacetylation and OES localization for Plasmodium ookinete gliding and mosquito infection'. This paper depends heavily on western blotting and single blots are shown throughout, unquantified, usually stated 'representative of 2 independent experiments'.

The only experiments in our manuscript that are reported just in duplicate are the timecourses (Fig 2A-B, E and 3E-H). Importantly, we report only trends here, without statistics, because this is essentially qualitative data for which statistical significance (between timepoints x and y but not x and z) would really add no useful insight. We set out to examine the time dynamics with which lactylation can rise and fall, not the exact magnitude thereof at every timepoint. (In fact, we would prefer to show such data unquantified, but universal quantification of western blots was requested in previous rounds of review.) Furthermore, timecourses of *Plasmodium* growth are affected by uncontrollable biological variability: the tightness of synchronisation is limited and the cell-cycle lengths can vary by several hours in different batches of human blood. These factors are less of a problem in systems like human cell culture, but they make exact reproducibility (and hence meaningful quantification) unrealistic here. Again, a selection of other high-impact publications shows that we have at least met the standard in the field for western blotting of timecourses:

- The original Nature paper (using timecourses in better-controlled human cells): Figures 3b and c are western blot data from timecourses quantified from just 2 experiments.

- A *Plasmodium* example, Nature 2025: 'Plasmodium blood stage development requires the chromatin remodeller Snf2L': This paper shows several timecourses where one histone western blot is shown, representative of just 2 experiments (unquantified), e.f. Fig S3a; S2h, etc.

Uncropped images of Western blots should be shown in supplementary data.
All uncropped western blots are now provided in the source data file.

2. Furthermore, it needs to be explained more clearly how many biological replicates per experiment were performed. Was only one biological replicate analyzed per experiment? This is not sufficient.
We have checked carefully, and the number of independent experiments for western blots was stated in every figure legend. We have now normalised the legend texts so that 'n=4/3/2' appears in a consistent format in every legend.

3. The quantification of the data in most Western blot experiments are normalized to the controls. However, the way this is done is not OK, since all controls appear normalized to themselves (resulting in a value of one), which does not allow to evaluate variability between controls, and which results in incorrect statistical evaluation. Normalisation should be performed to the mean of several biological replicates of the control condition. Also the individual control datapoints should be normalized to the mean, allowing to visualize and evaluate their variability.
When quantifying several independent western blots from independent experiments and then comparing them, it is necessary to set the control at time-0 to a consistent point (i.e. 1). Indeed this does hide the potential variability between controls at time-0. However, such variability is inevitable, and not biologically meaningful, because every different western blot will have a different level of background and exposure, which means that visual variation between blots made on different days from different experiments is not, per se, meaningful. Only the differences between lanes within a western blot are meaningful. (To avoid this issue, one must conduct and expose one single blot covering all the biological replicate experiments together, and loading identical amounts of protein from every experiment. But it is simply not possible to fit 30-40 lanes on one blot.)
See for more discussion of this 'The Design of a Quantitative Western Blot Experiment' (2014) https://doi.org/10.1155/2014/361590).

Furthermore, why is this normalization performed in most experiments, but not in fig S4?
In Fig S4, there were only 6 histone preps for M/S, made in parallel, so all conditions (3x –lactate and 3x +lactate) could be blotted in parallel on a single blot. Hence the above-explained normalisation was not necessary.

4. The specificity of the used antibodies need to be documented. Can these Abs fully differentiate between e.g. acetylation and lactylation on these plasmodial histones? This needs to be documented and relevant controls need to be provided. Are these antibodies raised against Plasmodium histones, or against which species? The mass spec data show that these modifications are specific between different Plasmodium species, so how to understand the specificity of these antibodies? This needs to be clarified.
Concerning the specificity of the antibodies for lactyl versus acetyl, this has been thoroughly validated by the producer (PTM Biolabs). In the case of the Pan-KLa antibody, it was raised against lactyl-lysine alone (context-free): therefore, using it on histones from any species including *Plasmodium* in equally valid. In the case of the histone-peptide antibody against H4K12La, this was raised by PTM Biolabs against the mammalian histone H4 peptide sequence. Verification re. *Plasmodium* is not, however, needed because histone sequences are near-identical across most eukaryotes: an example alignment of the human and *Plasmodium* histone H4 is shown below, with complete conservation of the entire tail and every lysine residue. (Our mass spectrometry data further confirmed that H4K12 was lactylated (i.e. + 72.02 Da) in *Plasmodium*, as it was in human.) Accordingly, these lactyl-histone antibodies have already been used in published work on at least human, mouse, *Toxoplasma* and *Trypanosoma* histones, without cross-validation for every species.

```
  1 MSGRGKGGKGLGKGGAKRHRKVLRDNIQGITKPAIRRLARRGGVKRISGL     50
    |||||||||||||||||||||:||||||||||||||||||||||||||||
  1 MSGRGKGGKGLGKGGAKRHRKILRDNIQGITKPAIRRLARRGGVKRISGL     50

 51 IYEETRGVLKVFLENVIRDAVTYTEHAKRKTVTAMDVVYALKRQGRTLYG    100
    ||||.||||||||||||:|::.||||||||||||||:||:||||||||||
 51 IYEEIRGVLKVFLENVIKDSIMYTEHAKRKTVTAMDIVYSLKRQGRTLYG    100

101 FGG    103
    |||
101 FGG    103
```

5. What is the rationale to expose early trophozoites to lactate? It would be more logical to expose schizont stages, which is the sequestering stage and most likely exposed to the highest concentrations of lactate?

Parasites sequester *in vivo* from the trophozoite stage onwards (only rings, younger than ~24hpi, are seen in the peripheral circulation). We started our lactate exposures at the trophozoite stage for this reason, and also because this is the start of the most metabolically active (i.e. lactate-producing) stage.

Minor comments:

Introduction: there are many more possible causes of hyperlactatemia besides parasite lactate production and anaerobic respiration in host tissues due to parasite sequestration. This should be briefly mentioned.

We have added this point to the introduction (second paragraph).

Fig 1D contains a strange outlier. More biological replicates are required.

We agree that there is a potential outlier in this figure, but we would argue that this represents real variation between the three independent replicate experiments. Adding another replicate would not obliterate the outlier, and a single dataset should not be excluded without any rationale. Note that this figure reports that there is NOT a statistically significant change in levels of H4K12La in *P. knowlesi*, and including or excluding the 'outlier' does not impact this conclusion.

Correlation analyses: p-values have to be indicated. Please also add units (or fold change?) to Y axes of correlation graphs.

We believe this refers to the plots in Fig 1E and Fig S1: we have added p values for the Pearson correlation coefficients stated in these legends.

The y-axes cannot have units because they are ratios (of western blot intensities, KLa / H4).

The authors indicate that P. berghei ANKA does not cause hyperlactataemia, but figure S1 shows values up to 15 mM. Please clarify.

We did not state that *P. berghei* ANKA does not cause hyperlactataemia, but that it does not do so in a consistent manner that is linear with infection progression ('not all rodent malaria models lead consistently to hyperlactataemia and in the *P. berghei* ANKA strain, which does not…').

This was shown by co-authors Cunnington *et al.* in doi.org/10.7554/eLife.70763:

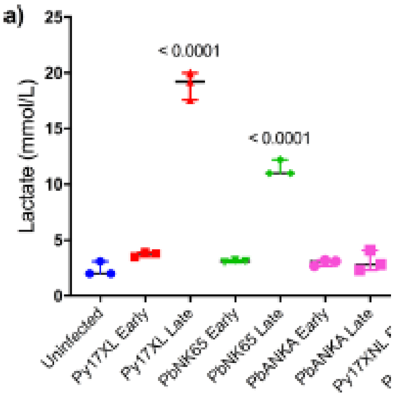

Lactylation appears still present 6 hours after lactate withdrawal in Pk but not in Pf, please discuss. Or is this the result of insufficient sample numbers? See comments above.
We have added to the results text a comment concerning the relative rates of delactylation between the two species (new lines 180-182).

Line 225: when stating a trend, it is essential to show the p-value.
Since the statistical test here was ANOVA with Tukey HSD post hoc test, there is not a single p-value but rather a matrix of p-values. However, we have now stated in the legend that the lowest p value within the trend mentioned was 0.21 (new line 261).

Fig 4: please clarify what is HA.
We have added this to the legend ('heterochromatin protein 1 with a haemagglutinin (HA) tag') (new line 295).

Line 444: Hyperlactatemia is very common in severe malaria patients. The authors are encouraged to also analyse the lactylation in patient samples, this would strongly improve the study.
We agree that it would be very interesting to examine histone lactylation in human patient samples of *P. falciparum*, and we have made grant applications to fund the requisite human-patient studies in malaria-endemic populations. However, human clinical studies are a large undertaking, beyond the scope of this initial study.

The ChIP experiment in figure 8 is hard to understand and should be better explained. Also explain clearly which 5 genes, how were these chosen?
The selection of genes is explained in the text ('We used chromatin immunoprecipitation (ChIP) to check whether a selection of *P. yoelii* genes <u>that were differentially expressed</u> in hyperlactataemic hosts were also differentially marked with lactyl lysine (fig 8C, fig S12C).)
However, we had not originally named all the chosen genes in the text (stating only 'These genes encoded factors involved in gametocytogenesis (AP2G), metabolism (LDH, PFK), etc.') We have now added the names of all 5 genes, which were previously only in the Methods, in full to the main Results text (new lines 552-555).

Why using different ways of lactatemia measurement for the two murine parasites, are these fully comparable? Are both specific for L-lactate?
All quantifications are for L-lactate (D-lactate is anyway a very minor fraction of the chemical in a healthy mammal, since it is only produced as a by-product of gut microbiota fermentation or methylglyoxal metabolism, and it scarcely appears in the blood).
Two methods were used because the *P. yoelii* experiments were conducted by co-authors at Imperial College London, whereas the *P. berghei* experiments were conducted by different co-authors in Cambridge (simply because of the availability of the two rodent models at the two locations). The clinical 'Lactate Pro 2 meter' was available at one location (Imperial College London) but not at the other, so a biochemical assay was used instead when the meter was not available.

<u>Reviewer #2:</u>
In their manuscript "Histone lactylation: a new epigenetic mark in the malaria parasite Plasmodium", Jabre et al. describe histone lactylation as an epigenetic mark in malaria parasites for the first time. The manuscript is generally of great interest and technically divided into two sections. Histone lactylation is first investigated using quantitative Western blotting. This involves investigating the dynamics of histone lactylation in blood stage parasites of P. falciparum and P. knowlesi upon addition of lactate and the decrease in histone marking upon withdrawal of lactate. Subsequently, the histones and non-histone proteins that are lactylated are identified and categorized using mass spectrometry. Furthermore, it is described that lysines that are lactylated are usually also acetylated, and it is suspected that acetyltransferases also transfer lactate to histones.

My comments are of a more general nature:

1. Introduction: The introduction does not read well. It seems very generic and incoherent. It should be revised to provide a logical introduction to the topic of the study.
We have added some extra context to the Introduction.

2. The results section should briefly describe the respective method used. For example, in line 99ff, it is not clear that this is quantitative Western blotting.
We have checked the text and added wherever necessary a statement of which method was used for each section.

3. The blood stages were treated with lactate for 12 hours (or 8 hours). Why wasn't the treatment time shorter?
In the timecourse experiments (Fig 2), we did test shorter times as well (2h, 6h etc.) to see how quickly lactylation could occur. However, for the initial assessments in Fig 1, we wished to see a robust, statistically-significant change, if such could be induced. Therefore we opted for a relatively long exposure, throughout most of the trophozoite stage, which is the stage of maximum parasite growth and metabolic activity.

4. It is generally unclear to me whether sufficient biological replicates were performed in the various experiments (especially in the mass spec experiments). This should be better described, or if the biological replicates are missing, they must be added.
We performed the M/S in triplicate, as is standard in the field (after an initial pilot experiment on mixed-stage parasites, which was conducted singly). We have ensured that this is clear in the text: (quoting from the start of the M/S section, line 301: 'For a comprehensive view of the histone lysine residues that can be lactylated in both *P. falciparum* and *P. knowlesi*, we used liquid chromatography-tandem mass spectrometry (LC-MS/MS). Trophozoite-stage parasites were either exposed or not exposed to 25mM lactate and their histones were acid-extracted from chromatin in biological triplicate. In a single pilot experiment, histones were also extracted from mixed stage cultures (+/- 25mM lactate), enabling detection of KLa sites in all stages of the asexual cycle.)

5. The word "crashing" is very colloquial. If possible, it should be replaced.
Although colloquial, we think that this is common term in the field. We placed it in quote marks to denote that it is colloquial, and then explained it in the text, together with a reference to a full paper that discusses the phenomenon (quoting the text, line 189: 'crashing' (the term for parasite death due to high parasitaemia and exposure to spent media [16]))

6. It is described how the same lysines in the histones can be both lactylated and acetylated. If I understand correctly, both marks are present on the lysine at the same time. However, it is unclear to me what this looks like at the molecular level. This should be visualized.
Many of the same lysines can be –La or –Ac at a population level, but not on the same molecule at the same time. This is because the same linkage would be used by either modification
We have now added a schematic showing this (Fig 1A), and have also clarified this in the text (line 343): 'Since the same lysine linkage is used, the same site on the same histone molecule cannot be simultaneously modified with both, but histones within a population of molecules can be modified with either.'

7. Acetylation reversal occurs via HDACs. Which enzyme reverses lactylation?
This is an area of active research in this very new (<6 years old) field, in human cells. We are actively working on this in *Plasmodium* but as yet there is no literature outside the mammalian system. In mammalian cells, several HDACs appear to be relevant, see example papers:
- 'Sirtuin 1/sirtuin 3 are robust lysine delactylases and sirtuin 1-mediated delactylation regulates glycolysis'. Du et al., iScience (2024) doi.org/10.1016/ j.isci.2024.110911
- 'Class I histone deacetylases (HDAC1–3) are histone lysine delactylases'. Moreno-Yruela et al., Sci. Adv. 8, eabi6696 (2022)

8. To obtain more information about whether histone acetyltransferases are responsible for lactylation,

knockout studies should be conducted, either using chemical HAT inhibitors or existing HAT knockouts. The use of HDAC inhibitors such as TSA would also be interesting. Overexpression studies could also be conducted for HATs.
We agree, and we have this work actively underway. However, since a thorough study of writer and eraser enzymes for histone lactylation requires creating conditional knockdowns for *at least* 7 *P. falciparum* enzymes, plus the full characterisation of downstream epigenetic effects (and since a single such knockdown frequently supplies data for an entire paper in this field), we take this as a suggestion for a subsequent paper, not for inclusion in this one.

9. Which enzyme lactylates the non-histone proteins?
This is not fully known, even in model systems, but alanine t-RNA synthetase (AARS), which is cytoplasmic as well as shuttling to the nucleus, plays a role in human systems (see references to this enzyme in our paper): we have this under analysis, and our preliminary data suggest that AARS plays a role in *Plasmodium* as well.

10. Line 295: The text should describe which KLa sites were discovered, or at least mention the common KLA sites between P. falciparum and P. knowlesi.
We have listed the sites in the text now (line 313-316), as well as showing them on the figure.

11. It has been shown that both the genes of sexual commitment markers and the proteins themselves (AP2-G, MDV1) are lactylated. Therefore, it should be investigated whether the addition of lactate and histone lactylation has an influence on sexual commitment. Does lactate trigger or inhibit sexual commitment?
Lactate has been published to enhance commitment, as referenced in our introduction (doi: 10.1128/IAI.00635-20). We are currently working on the mechanistic pathway re GDV1/AP2-G.

12. Lines 520ff: The five genes should be named.
We have done this (new lines 552-555).

Minor:
Line 267: It is HP1
Thank you: the typo (I for 1) has been corrected

Lines 375/376: A word appears to be missing at the end of the sentence (after "were").
We have added ‘were lactylated’

Figure caption Fig. 8. It must be made clear here that previously published data sets from [14] were analyzed.
We have added this now to the legend, as well as the results text where it was already stated.

Reviewer #3:
Here, Jabre et al. investigate the relatively unexplored epigenetic modification of lactylation and how it may impact the transcriptional programme of the malaria parasite as the infection progresses. Hyperlactatemia is a clinical manifestation during Plasmodium infection, and this study examines the novel concept that the parasite senses this metabolite and in a very direct way, by histone lactylation, with the implication that it may alter gene expression levels. Proteomics has also identified lactylation on non-histone proteins, suggesting a potential role for this modification beyond histones, as previously reported in higher organisms.

The study first shows an apparent increase in histone lactylation by supplementation of lactate in the media, as well as a correlation in vivo between lactylation and blood lactate levels. Dynamics of lactylation in both P. falciparum and P. knowlesi were examined using a pulse, showing relatively rapid, but transient, modification. Using proteomics, the authors then mapped the specific histones and residues that are modified in both P. falciparum and knowlesi, allowing comparison of species-specific differences. Finally, the authors aim to correlate expression of genes at late stages of infection

when lactate levels are elevated, with their association with lactylated histones. Overall this study describes a new epigenetic mark with implications for gene expression during different phases of malaria infection, and would be of broad interest to the community.

The authors may want to consider the following points for revision.

Main points:

1) Figure 8C shows a crucial piece of data that could be explained in more depth, as it aims to provide the connection between histone lacytlation and alterations in gene expression. If I've understood correctly, the authors first analyzed previously published data (Georgiadou et al., 2022) on gene expression from an in vivo P. yoelii infection, deriving a gene set of differentially expressed genes upregulated in late-stage infection (and thus potentially associated with higher lactate levels). They then perform their own experiment using ChIP of lactyl-histone sites in three infected mice. However, the transition from analysis of the previous work to their own experiment (page 23, line 517) could be more clearly delineated, as this was not immediately clear.
The reviewer understands correctly, and we have clarified it further in the text (new lines 544-547).

Similarly it was not immediately obvious that the three different lactate levels shown in the figure legend of Fig.8C were obtained by sampling from the infected mice. Were these harvested at different times (as with the Georgiadou et al. paper) or was this a fortuitous result that a range of lactate levels were obtained,
The mice were sacrificed over 2 successive days, after measuring blood lactate from the tail vein to get an idea of whether the mice currently had high or low lactate, and thus to obtain parasite samples from both states.
and did that also correlate with parasitemia.
Yes, parasitaemia and blood lactate correlated broadly (R^2 0..72, see below).

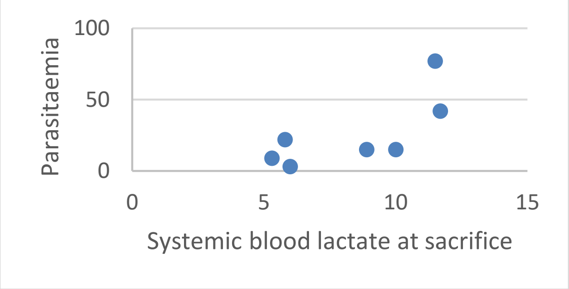

The text states that "ChIP was used to determine whether a selection of P. yoelii genes differentially expressed in hyperlactataemic hosts were also differentially marked with lactyl lysine." However, this may give the impression that ChIP was performed on all deregulated genes, when it was conducted on five genes. It was not clear why these five genes were selected, nor how many were upregulated versus downregulated. Including ChIP analysis for a broader set of genes from both upregulated and downregulated categories would strengthen the conclusions.
We have made it clearer in the text that this was a relatively restricted gene-specific ChIP experiment. Concerning how the genes were selected, we first selected 5 genes that were strongly regulated, and that had known roles (e.g. LDH, PFK). Upon ChIP, we found all 5 of them to correlate in their levels of lactyl-chromatin vz host blood lactate (albeit with a broad range of fold changes). We then selected a somewhat larger set of 9 genes whose transcription was NOT regulated, and found a clear pattern – beyond any gene-to-gene variability – that these showed no correlation between chromatin lactylation and host blood lactate. Although this ChIP study was relatively small in scope, the result seems clear. No cherry-picking was done (that is, no genes were excluded or included after seeing the ChIP results.)

2) For the histone lactylation measurements in Fig. 2, it wasn't clear which of these were statistically significant? Was testing performed?

See response to Reviewer 1. From the timecourses in Fig 2, we do not report statistics because this is essentially qualitative data for which statistical significance (between timepoints x and y but not x and z) would not add much useful insight. We set out to examine the time dynamics with which lactylation can rise and fall, not the exact magnitude thereof at every timepoint. (If statistical testing *is* conducted, a few individual data points are different with a p value below 0.05 – for example, in Fig 2A, the 6h timepoint –lactate differed from the same timepoint +lactate. However, most points in the whole group did not show such a difference under ANOVA.)

3) Given elevated parasitemia in P. falciparum was shown by the authors to yield higher levels of lactate in the media, it would be informative for the in vivo P. yoelii experiment shown in Fig. 1F to know if the higher lactyl-histone modifications also correlated with parasitemia, if this is known.
This was true, although there was a somewhat lower correlation (R^2 0.48) between parasitaemia and histone lactylation than between parasitaemia and blood lactate.

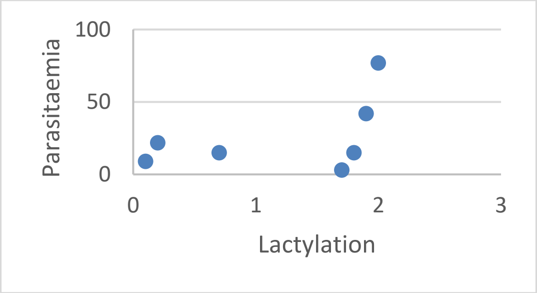

<u>Minor points</u>

4) In Fig.2C, the graph shows the parasite culture 'crashing' at only ~0.5% parasitemia – is this an error in labelling? It would be surprising if this occurred at such a low parasitemia.
We appreciate the spot: there is a 10x scaling error on the right-side Y-axes, and we have corrected the plots.

5) Supp. Fig 2 – it is unclear whether B or C shows the lactate-treated experiment, and the figure legend does not describe panel C.
We appreciate the spot of this omission, we have clarified the legend and also added labels to the graphs in the figure.
