# Supplementary material for: Histone lactylation: A new epigenetic mark in the malaria parasite Plasmodium
Source: PLoS Genet. 2025 Dec 19;21(12):e1011991. doi: 10.1371/journal.pgen.1011991 (PMC12742760; doi:10.1371/journal.pgen.1011991)
Supplement: Attachment — Submitted filename: Response to Reviewers.docx [file pgen.1011991.s023.docx]

Point-by-point responses to the peer reviews are provided below in red text.

Reviewer #1:

Although some of the issues have been solved, two main problems in this paper remain. The lack of reasonable numbers of datapoints remains a major problem, as well as the way the Western blots have been normalized.

Major comments
1. The authors still do not provide a sufficient number of datapoints per condition. 2 / 3 /4 datapoints per conditions is not sufficient. Essentially the whole paper appears based on such low numbers of datapoints, this is not OK. The fact that Western blot is a semiquantitative methods is not a good reason to validate such low sample numbers, semiquantitative methods may rather require extra confirmation. Referring to other papers in which such low sample numbers might have been used is equally not a good rationale. I agree with the necessity to quantify Western blots by e.g. densitometry, and the main reason for such quantification is to apply statistics on a reasonable number of samples, enabling to draw solid conclusions. With such low sample numbers and considerable variation in many of the graphs, the conclusions are not sufficiently backed up by the data.

To meet the reviewer’s requests, we have now added extra biological-replicate blots to each lactate titration, giving 6 biological replicates in total, instead of the previous 3-4. (Reviewer#1’s previous review stated ‘an absolute minimum of 5 to 6 datapoints per condition (and per time point) is required’, so we have now boosted the replicate numbers to the requested 6.) See the revised Fig 1B-E and Fig 3A-D. These additional replicates all support our original conclusions.

We have removed the timecourse data entirely from Figures 2 and 3. Although it added useful detail to the story, its removal does not materially affect our conclusions and it would have been impractical to provide as many as 6 biological replicates for each timecourse as well. Performing an extra 4 replicates, on top of the existing 2, for the timecourses in Fig 2A-B and 3E-H, would have required a volume of work that was impractical within a 2-month revision deadline (i.e. 96 additional western blots, on top of the 48 already being added for Fig 1B-E and Fig 3A-D). We have therefore opted to remove this strand of data instead, preserving all the manuscript’s major conclusions.

2. The problem of normalization of the Western blot data has not been solved. In particular, controls are normalized to themselves, resulting in the problem that all controls get the value of 1. This is statistically not OK, as it does not permit to evaluate the variation in the control condition. The authors refer in their reply to ‘The Design of a Quantitative Western Blot Experiment’ (2014) <https://doi.org/10.1155/2014/361590>). However, they do not apply at all the normalization method proposed in this paper, which suggests to normalize the loading controls to a (reference) control sample that is loaded onto lane 1 of all blots. Literally from this paper: “control is typically a pooled homogenate from all of the samples in a given study aliquoted into multiple tubes to permit the loading of a fresh control sample in lane 1 of each study blot”. In table 2 of that paper an example is given, in which a clear standard deviation (different from 0) is shown. Alternatively, 3 or 4 control samples must be loaded on each gel, and the data normalized to the mean of these controls.

We accept that normalising the controls to 1 throughout does prevent evaluation of variation between them. Although this is not uncommon in the literature, we have now changed the analysis to avoid this. Data are no longer normalised to 1, so the full variation between the now-6x replicate experiments can be seen. Data for each replicate experiment are now presented simply as the ratio between control H4 and modified histone in each blot.

Reviewer #2:

I am satisfied with the responses to my comments and have no further comments.

Thank you.

Reviewer #3:

The authors performed a number of textual modifications in response to the critiques. They did not choose to include additional genes in the ChIP analysis (Q3), however I’ll accept that their claims are largely restricted to those genes that were analysed.

For the question on replicates (Q2), which was also raised in one way or another by the other reviewers, the authors indicate that they are only showing qualitative trends (e.g. across timepoints). However, that’s not quite how the text reads.

For example, for Figure 2 (line 177-180): “We repeated these experiments in P. knowlesi (fig 2B): again, the histone lactylation signal was inducible across the trophozoite stage of the cell cycle, while background levels of lactylated histones were low, variable, and not clearly cell-cycle-regulated in this species (fig 2B: grey bars)”. This implies addition of lactate is inducing a quantitative increase in lactylated histones relative to the no-lactate condition. But, for example at timepoint 6/MT, one datapoint is the same as the no-lactate control and one is high.

Similarly for Figure 3E-H (line 246-248): “Across the normal cell cycle, fluctuations in acetylation were less than 2-fold, but after exposure to 25mM lactate, histones were, again,
additionally acetylated, particular on H4 in P. falciparum.” The claim that they are additionally acetylated relative to the control is a quantitative comparison, but the differences shown are small that it is hard to know if these are biologically meaningful.

As per reviewer 1, we have removed all timecourse data from the manuscript, and hence also removed any text that refers to this, such as that quoted above.

In response to reviewer 1, the authors note that the original lactylation Nature paper quantified blots from two independent replicates (e.g. Fig3b,c). However, those were also done in technical triplicate, which should make the data more robust.

This point is related to literature-precedents, not to the current manuscript.

However, we would question how much more robust this could actually make the Nature paper’s data. That legend simply stated *‘Histone acylations were analysed by immunoblots using whole-cell lysates. ImageJ was used for quantification; n = 3 technical replicates. Data represent two independent experiments.’* This very brief legend obscures the question of whether densitometry was simply repeated 3x on the same blots, or whether the same blots (from 2 just biological experiments) were repeated 3x and quantified once each. In even the latter case, this would require less work, and also generate much less variability, than performing 6x independent biological experiments, which is the markedly higher standard we have now met here.

Thus it seems that either additional replicates would be required to substantiate the claims of lactate induction (potentially just at a single timepoint if not a whole timecourse) or a more cautious interpretation is required.

As suggested, and as per Reviewer 1, we have added extra data on the single timepoint experiments, bringing the total biological replicates to 6 (at each of 4 lactate levels): our conclusions remain essentially unchanged.
